# Systematic Review Comparing Open Versus Minimally Invasive Surgical Management of Intradural Extramedullary Tumours (IDEM)

**DOI:** 10.3390/jcm14051671

**Published:** 2025-03-01

**Authors:** Asfand Baig Mirza, Ariadni Georgiannakis, Feras Fayez, Pak Yin Lam, Amisha Vastani, Christoforos Syrris, Dale Darbyshire, Kevin Tsang, Cheong Hung Lee, Amr Fahmy, Zaher Dannawi, Jose Pedro Lavrador, Irfan Malik, Gordan Grahovac, Jonathan Bull, Alexander Montgomery, Ali Nader-Sepahi, Taofiq Desmond Sanusi, Babak Arvin, Ahmed Ramadan Sadek

**Affiliations:** 1 Department of Neurosurgery, Queen’s Hospital Romford, Barking, Havering and Redbridge NHS Trust, Essex RM7 0AG, UK; 2 North East London and Essex (NELE) Spine Network, London E1 1FR, UK; 3 Department of Neurosurgery, King’s College Hospital NHS Foundation Trust, London SE5 9RS, UK; 4 Barts and the London School of Medicine, Queen Mary University of London, London E1 2DP, UK; 5 Imperial College Healthcare NHS Trust, London W2 1NY, UK; 6 GKT School of Medical Education, King’s College London, London WC2R 2LS, UK; 7 AxIOM Neuromonitoring Ltd., London W1W 5DT, UK; 8 Department of Trauma and Orthopaedic Surgery, Mid and South Essex NHS Foundation Trust, Essex SS0 0RY, UK; 9 Department of Neurosurgery, The Royal London Hospital, Barts Health NHS Trust, London E1 1BB, UK; 10 Wessex Neurological Centre Neurosurgery, Southampton General Hospital, University Hospital Southampton NHS Foundation Trust, Southampton SO16 6AQ, UK

**Keywords:** IDEM, systematic review, minimally invasive, laminectomy

## Abstract

**Background/Objectives**: Intradural extramedullary (IDEM) spinal tumours are relatively rare and predominantly benign. Gross total resection (GTR) has been demonstrated as an effective treatment, with increasing evidence supporting the use of minimally invasive techniques to achieve GTR. This study reviews the current surgical management options for IDEM tumours and their outcomes. **Methods**: A systematic literature search without meta-analysis was conducted by two independent reviewers in December 2024. The population of interest comprised patients who underwent surgical treatment for IDEM tumours. Outcomes assessed included the extent of resection, postoperative neurological function, and complications. **Results**: Fifty-seven articles met the inclusion criteria, providing data on 4695 IDEM cases, of which 3495 were managed through open surgery and 750 via minimally invasive surgery. The extent of resection was high, with a mean GTR > 90% across studies. Open laminectomy and unilateral minimally invasive hemilaminectomy were the most common surgical approaches. Complications, such as cerebrospinal fluid leaks, were less frequent following minimally invasive procedures vs. open surgery (11.1% vs. 14.3%). Minimally invasive surgery also led to improved postoperative functional outcomes (mean McCormick score change −1.30 vs. −0.64) and a lower recurrence rate (1.4% vs. 10.0%). **Conclusions**: Whilst open surgery yields acceptable rates of resection and neurological improvement, there is growing evidence that minimally invasive surgery can achieve comparable, if not superior, rates of resection with fewer complications, leading to lower costs and shorter hospital stays.

## 1. Introduction

Spinal tumours are rare, accounting for approximately 5–10% of tumours in the central nervous system [1]. Typically, spinal tumours are further categorised based on their location in the spine: intradural extramedullary (IDEM), intradural intramedullary, and extradural. Most spinal tumours, almost 70%, are found in the IDEM space [1,2].

IDEMs can be broadly divided into three main tumour types: meningiomas, nerve sheath tumours (schwannomas and neurofibromas), and filum terminale ependymomas. Several other IDEM tumours include dermoids, epidermoids, teratomas, and lipomas; however, these combined only account for approximately 5% of IDEMs [3,4]. Conversely, meningiomas and schwannomas account for over 50% of IDEM tumours [5]. There is a debate as to whether meningiomas or schwannomas are more commonly observed, with varying reports in the literature [3,6,7].

Symptoms and presenting complaints of IDEMs can be variable. Initial presenting symptoms can be non-specific, making it difficult to achieve early diagnosis [7]. This includes back pain that radiates to the limbs, thermal analgesia, and muscle weakness [8,9]. Over time, more specific neurological symptoms may arise due to compression of the spinal nerves and or the spinal cord [6,8,10]. The specificity of these symptoms, however, largely depends on the location of the tumour around the spinal cord, as well as the vertebral level and type of tumour. Tumour location also largely dictates the urgency of the treatment, with tumours directly compressing the spinal cord requiring prompt intervention [11].

IDEM tumours are generally managed surgically, with most surgeons aiming to have a gross total resection (GTR). Due to the benign nature of most IDEM tumours, GTR results in favourable outcomes with low recurrence rates [12,13]. Despite this, the literature has considerable variance concerning the optimal approach. Traditional open surgery is useful in achieving a high GTR and minimal long-term neurological impairment. On the other hand, minimally invasive surgery may be more beneficial in reducing the impact on soft tissue and midline structures, as well as reducing the duration of in-patient stays [14]. Predominantly, the question of open versus minimally invasive surgery remains uncertain. In addition, many authors report using intraoperative neurophysiological monitoring (IONM) in IDEM surgery, as it has proved helpful in other spinal tumours. However, there is lacking evidence of its viability in IDEM tumours specifically.

A systematic review has hitherto not been conducted in the surgical management of IDEM tumours. As such, we decided to conduct this systematic review, comparing outcomes of IDEM tumours in open versus minimally invasive surgery.

## 2. Materials and Methods

The preferred reporting items for systematic reviews and meta-analyses (PRISMA) guidelines were followed in this study [15], and a protocol was registered on PROSPERO (ID: CRD42025631746). A systematic search was performed in December 2024 on the EMBASE and MEDLINE databases. Databases were searched for keywords “IDEM”, “EIST”, “intradural extramedullary”, or “extramedullary intradural” since inception. A filter was applied to only show studies in English, with abstracts available, and conducted in humans. Additionally, we performed reference harvesting of previous reviews, for any articles that may have been missed. After removing duplicates, titles and abstracts were screened by two reviewers (AG and ABM). Any conflicts were resolved through discussion between at least three authors. The inclusion criteria for eligibility consisted of articles that focused on adult IDEM tumours and provided information on surgical outcomes. Articles that included all spinal tumour types were only included if they separately isolated findings of IDEM tumours from other tumour pathologies. In addition, articles were required to have more than five IDEM patients in their study. Any paediatric cases, non-surgically managed tumours, or articles with five or fewer patients were excluded.

Articles that passed the screening processes had their full texts reviewed by two reviewers (AG and PYL). The absolute minimum requirement for inclusion was articles that specified results for IDEM tumours and provided a rate of total resection and information on surgical technique. Any ambiguity in meeting these minimum requirements resulted in exclusion from the study. The article could be included in the systematic review if the paper was a cohort study comparing two surgical techniques. However, data were extracted from two cohorts rather than combining the results.

Once the article selection had been finalised, the required information was extracted by two reviewers (AG and PYL). Firstly, we gathered general information from the paper, including the year of publication and the number of patients included in the study. Next, basic demographics were extracted, including mean age or age range, gender ratios, presenting symptoms, and duration of symptoms. Tumour information included pathological diagnosis, location, and position. As surgical management was the primary focus of the systematic review, we gathered information on surgical approach, rate of resection, use of surgical aids, complications, and outcomes. Finally, follow-up data were analysed, including rates of recurrence and mortality. During this stage, we also analysed papers for risk of bias using the Newcastle–Ottawa Scale. Any paper deemed to have a high risk of bias was excluded.

After data extraction, papers were divided into an “open surgery” group and a “minimally invasive” group. For this systematic review, open surgery was defined as operations where laminectomy, laminotomy, or laminoplasty was performed; minimally invasive surgery was defined as operations where hemilaminectomy was performed. The only exception to this was with retrospective cohort studies comparing open versus minimally invasive approaches where we followed the authors’ definitions.

Data were synthesised with both narrative and meta-analytical methods. A meta-analysis was performed in Python 3.13.2 DE United States to compare postoperative outcomes, such as the extent of resection, complications, and recurrence between the open and minimally invasive groups. Rates (%) were pooled from studies and used as the effect measure.

## 3. Results

**Figure 1:**
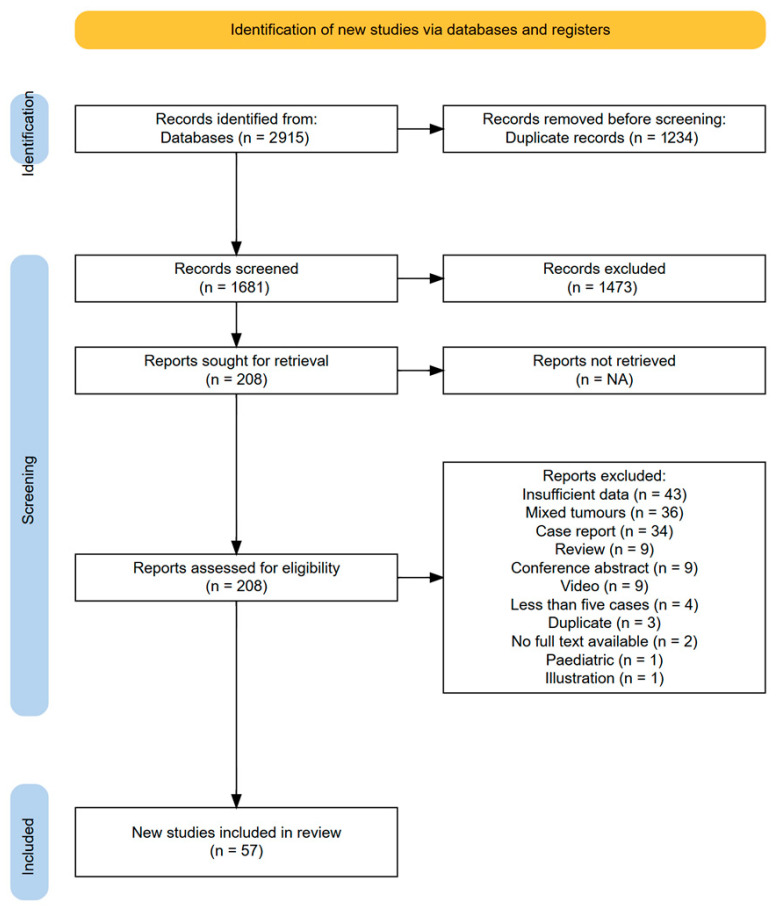
PRISMA flowchart of included studies [70].

A total of 57 articles met the inclusion criteria for analysis (Figure 1) [7,12,16,17,18,19,20,21,22,23,24,25,26,27,28,29,30,31,32,33,34,35,36,37,38,39,40,41,42,43,44,45,46,47,48,49,50,51,52,53,54,55,56,57,58,59,60,61,62,63,64,65,66,67,68,69]. As per our methods, seven papers were cohort studies comparing open versus minimally invasive techniques, and thus, data were extracted for these two separate cohorts of patients [26,28,30,33,46,49,58]. Once papers were split into open versus minimally invasive groups as per our definitions, this resulted in 37 sets of patients in the open surgery group [12,17,18,19,20,24,25,26,28,30,31,32,33,35,36,37,38,39,40,41,42,43,44,45,46,47,48,49,50,51,52,53,54,55,56,57,58], and 26 cohorts in the minimally invasive group [7,16,21,22,23,26,27,28,29,30,33,34,46,49,58,59,60,61,62,63,64,65,66,67,68,69]. This, therefore, provided 63 cohorts of IDEM patients overall, providing information about a total of 4695 cases, 3495 of which were managed via open surgery and 750 by minimally invasive surgery.

The risk of bias analysis revealed that 15 studies were of moderate risk and 42 studies were of low risk. No study had a high risk of bias.

### 3.1. Demographics

**Figure 2:**
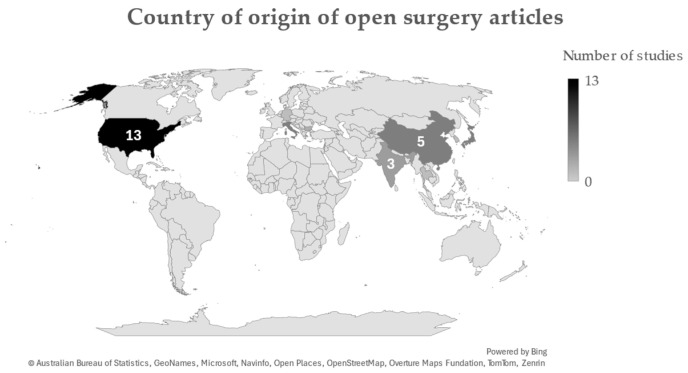

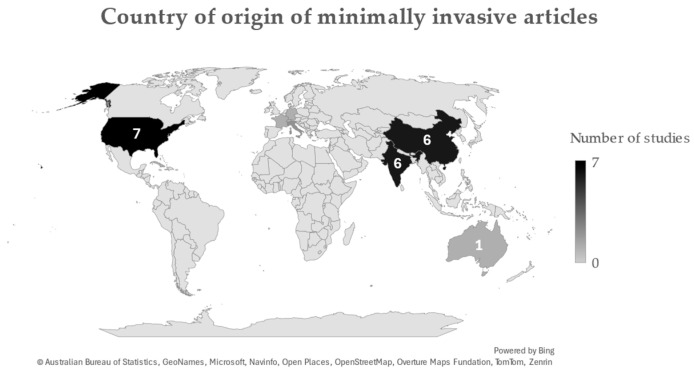
Countries of origin for studies included in this review. Figures were generated through Microsoft Excel.

A total of 54 out of 57 articles included gender information of their cohorts. Whilst male-to-female ratios differed considerably among some cohorts, combining all patients in this review yielded a total of 2133 male and 2623 female patients. Mean ages ranged between 35.5 and 74.6 years, with an overall average of 53.9 years. Most studies originated from the United States, China, and India (Figure 2).

### 3.2. Symptoms

Presenting symptoms were described in 36 patient cohorts. Pain was the most common symptom, most notably back pain (reported in 8 cohorts) and radicular pain (reported in 13 cohorts). Motor and sensory deficits were also common, reported in 28 and 23 cohorts, respectively, with considerable variation in severity from slight numbness and weakness to quadriplegia. Bowel and bladder dysfunction were also reported in 21 studies, and gait imbalance was reported in 5 studies.

Mean symptom duration (between symptom onset and surgery) was reported in 27 cohorts, ranging between 0 and 72 months, with an overall average of 11 months.

### 3.3. Tumour Pathology

Tumour pathology was reported in 61 cohorts, totalling 2656 patients. The most common tumours were schwannomas (49%), followed by meningiomas (35%) (Table 1). Other pathologies included ependymomas, neurofibromas, metastatic tumours, cysts, paragangliomas, and lipomas.

Additionally, tumour location was reported in 61 cohorts, including 36 open and 26 minimally invasive cohorts, totalling 3701 and 691 patients, respectively. In both open and minimally invasive cohorts, thoracic tumours were most common, followed by lumbar and cervical tumours (Table 2).

Tumour location was reported in 22 cohorts, including 15 open surgery and 7 minimally invasive surgery, totalling 695 and 369 patients, respectively. A variety of tumour positions have been described, including ventral, ventrolateral, dorsal, dorsolateral, and lateral tumours (Table 3). Notably, dorsolateral tumours were more common in minimally invasive cohorts, comprising almost half (*n* = 164, 44%) of cases compared to (*n* = 169, 24%) in open cohorts.

### 3.4. Surgical Approaches

All tumours included in this review were managed primarily through resection surgery. Rates of resection were high, with the overall mean GTR being over 90% in both open and minimally invasive cohorts. The lowest reported gross total resection rate was 55.8% [51].

**Figure 3:**
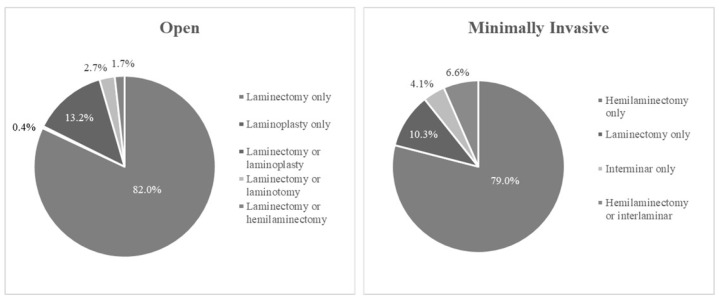
Surgical approaches in open and minimally invasive cohorts.

Various approaches were used to obtain access to the tumour, with open laminectomy and unilateral minimally invasive hemilaminectomy being the most common, conducted in 3235 and 591 patients, respectively (Figure 3). While most studies focused on either open or minimally invasive surgery, six papers performed retrospective cohort studies comparing the two techniques [26,28,30,46,49,58].

Additionally, there was considerable variation in the minimally invasive techniques used. Some were predominantly endoscopic, others microscopic, and some used tubular retractors (Table 4).

### 3.5. Postoperative Outcomes

#### 3.5.1. Open vs. Minimally Invasive Surgery

**Figure 4:**
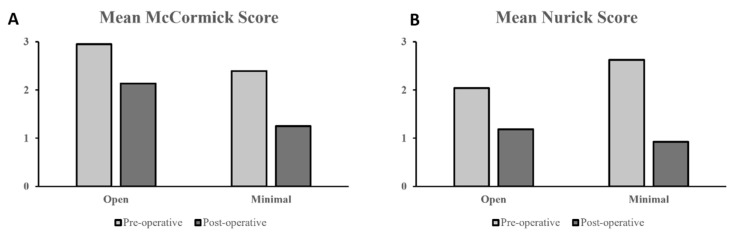
(**A**) Pre- vs. postoperative mean McCormick scores and (**B**) Nurick scores between open and minimally invasive surgery cohorts.

Most of the studies that reported postoperative outcomes used the modified McCormick scale and Nurick grading system. The McCormick scale was reported in eight open surgery cohorts and two minimally invasive surgery cohorts, while the Nurick system was reported in two open surgery cohorts and six minimally invasive surgery cohorts. Across both grading scales, minimally invasive surgery cohorts reported a noticeably more significant improvement in postoperative functional outcomes (Figure 4A,B). The mean change between pre-and postoperative McCormick scores was −0.64 for open surgery cohorts and −1.30 for minimally invasive surgery cohorts, while the mean change in Nurick scores was −0.86 for open surgery cohorts and −1.70 for minimally invasive surgery cohorts.

**Figure 5:**
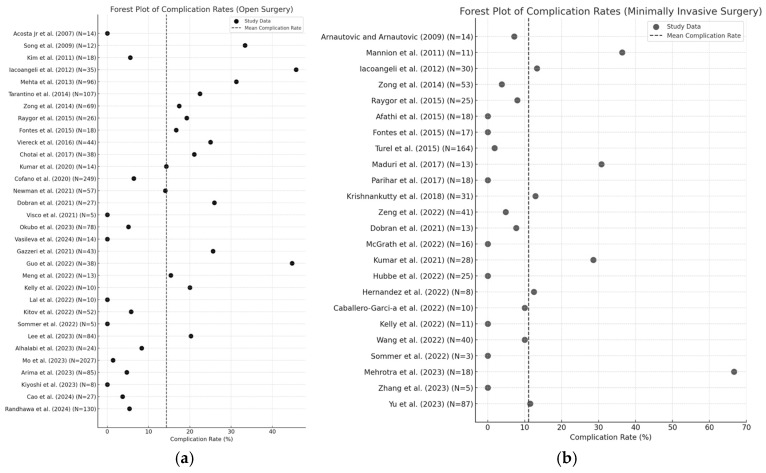
Forest plot illustrating complication rates in (**a**) open surgery and (**b**) minimally invasive surgery [7,12,16,17,18,19,20,21,22,23,24,25,26,27,28,29,30,31,32,33,34,35,36,37,38,39,40,45,46,49,50,51,52,53,57,58,59,60,61,62,63,64,65,66,67,68,69].

A total of 362 complications were reported across 45 patient cohorts, including 307 complications for 2147 patients (14.3%) in open surgery cohorts and 55 complications for 495 patients (11.1%) in minimally invasive surgery cohorts. Within the minimally invasive population, there were 13 complications for 66 patients (19.7%). Cerebrospinal fluid (CSF) leak was the most common, with 53 reported cases in open surgery and 10 cases in minimally invasive surgery. New-onset or worsening neurological symptoms were also commonly reported, including neuropathic pain, limb weakness and numbness, and focal neurological deficits. Other complications include site infections (17 cases), pseudomeningocoele formation (11 cases), haematomas (10 cases), spinal instability (13 cases), as well as vascular incidents such as deep venous thromboses, pulmonary embolisms and cerebrovascular (total 62 cases). An overview of complication rates according to open or minimally invasive surgery can be seen in Figure 5a and 5b. Mortality was reported in three open surgery studies [19,43,45], while no mortality cases were reported in minimally invasive surgery cohorts.

**Figure 6:**
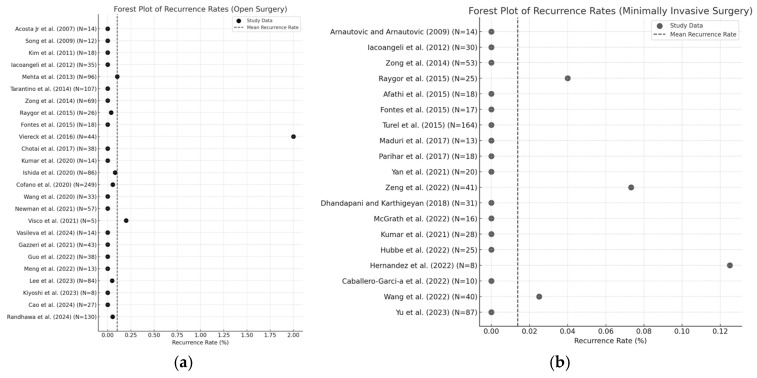
Forest plot illustrating recurrence rates in (**a**) open surgery and (**b**) minimally invasive surgery [7,12,16,17,18,19,20,21,22,23,24,25,26,27,28,29,30,31,32,33,34,35,36,37,38,39,40,45,46,49,50,51,52,53,57,58,59,60,61,62,63,64,65,66,67,68,69].

Mean lengths of follow-up varied between 1 month and 4 years, with an overall average of 24.3 months for open cohorts and 24.1 months for minimally invasive cohorts. The mean recurrence rate was considerably lower for minimally invasive cohorts (mean 1.4%) compared to open cohorts (mean 10.0%) (Figure 6a,b).

#### 3.5.2. Surgery With vs. Without Tubular Retraction

**Figure 7:**
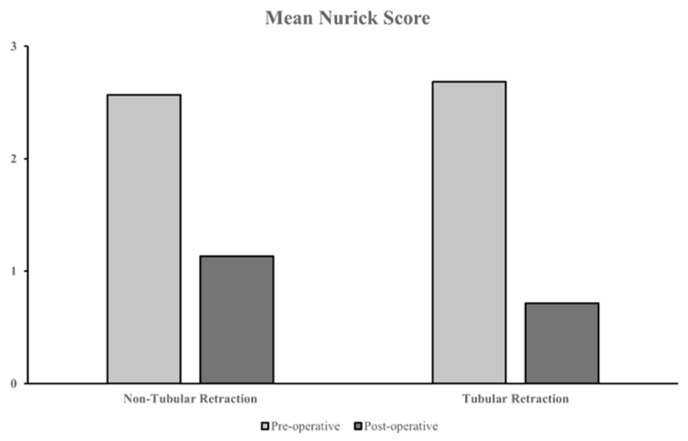
Pre- vs. postoperative mean Nurick scores between minimally invasive cohorts with and without tubular retraction.

Amongst studies involving minimally invasive surgery cohorts, 13 used tubular retraction and 10 did not use tubular retraction. These studies reported pre- and postoperative outcomes in four cohorts that underwent surgery with tubular retraction and five cohorts without tubular retraction. The majority of outcomes were reported using the Nurick grading system. Cohorts with tubular retraction reported a marginally more significant improvement in postoperative outcomes, with a mean Nurick score change of −1.97 compared to −1.43 without tubular retraction (Figure 7), and also reported marginally lower complication rates (mean 9.4% vs. mean 12.5% without tubular retraction). Nevertheless, one study reported an incidence where the wrong spinal level was exposed during tubular retraction surgery, resulting in the abandonment of the procedure [33]. The authors attributed this to difficulty localising the pathology using fluoroscopy in the mid- to upper thoracic spine, along with the limited scope of view and lack of access to explore surrounding levels when the tumour’s exact location was missed. No considerable difference was observed in recurrence rates (mean 1.83% with tubular retraction vs. mean 1.23% without tubular retraction).

#### 3.5.3. Endoscopic vs. Microscopic Surgery

**Figure 8:**
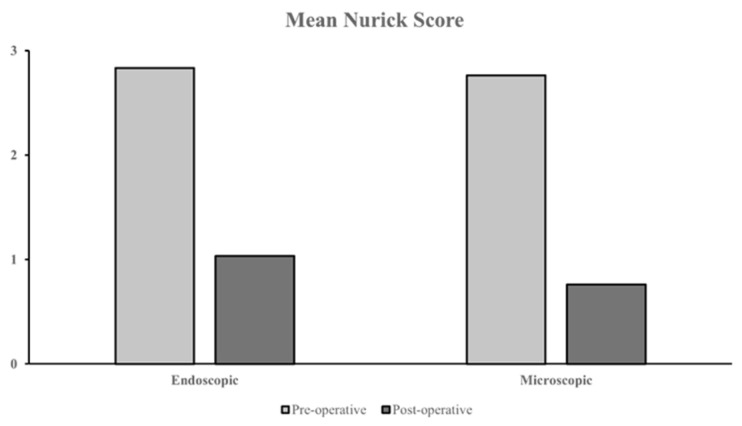
Pre- vs. postoperative mean Nurick scores between minimally invasive cohorts with endoscopic vs. microscopic surgery.

Additional comparisons were made between minimally invasive cohorts employing endoscopic and microscopic approaches, with or without tubular retraction. A total of 5 studies used endoscopic surgery, and 16 studies used microscopic surgery. Two studies each had two patient subgroups who underwent endoscopic vs. microscopic surgery [67,68]. Of these studies, two endoscopic and three microscopic cohorts reported pre- vs. postoperative outcomes using the Nurick grading scale. A marginally more significant outcome improvement was observed in the microscopic cohort, with a mean Nurick score change of −1.64 compared to −1.05 in the endoscopic cohort (Figure 8). A marginally lower mean complication rate was observed in the microscopic cohort (mean 9.0% vs. mean 15.3% with endoscopy). Recurrence was reported in four microscopic cohorts ranging between 2.5% to 14.3%, while no recurrence was noted in any endoscopic cohorts.

#### 3.5.4. Endoscopic Surgery with Tubular Retraction vs. Microscopic Surgery Without Tubular Retraction

Two cohorts underwent endoscopic surgery with tubular retraction (tubular endoscopic), and five cohorts underwent microscopic surgery without retraction (non-tubular microscopic), resulting in 21 and 305 patients, respectively. Demographics and tumour pathology data are shown in Table 5.

Postoperative functional outcomes were reported in one tubular endoscopic cohort and one non-tubular microscopic cohort using the Nurick grading system, with mean changes of −3.3 and −2.2, respectively. All non-tubular microscopic cohorts reported complications with a mean rate of 6.65%, and recurrences were reported in two cohorts with a mean rate of 8.4%. In contrast, no complications or recurrences were reported in tubular endoscopic cohorts.

### 3.6. Cost-Effectiveness of Open vs. Minimally Invasive Surgery

Although this was not an objective of this systematic review, we identified a study that performed a cost comparison analysis between open and minimally invasive approaches to IDEM tumours. Fontes et al. highlighted that although the cost of surgery and preoperative costs were not significantly different between the two surgical techniques, there were significant differences in postoperative care [26]. Due to the reduced number of complications occurring in the minimally invasive group, overall postoperative costs were significantly lower in the minimally invasive group. This also led to quicker discharges, reducing costs further by 24.5% overall. Zong et al. also reported this reduction in cost and shorter hospital stays with minimally invasive approaches, compared to open surgery [30].

## 4. Discussion

IDEM tumours, while a rare oncological entity, have seen improved prognoses over the years due to advancements in imaging, surgical techniques, and intraoperative monitoring [3]. However, secondary to the general rarity of spinal tumours, there remains a lack of published research specifically on IDEM tumours, underscoring the unique and important nature of this topic.

IDEM tumours are by and large slow-growing and benign, with the vast majority of cases categorised as WHO grade 1 tumours [71]. However, as the tumour grows, it can begin to compress the spinal cord; thus, surgical intervention is generally required in all symptomatic patients. Due to its benign nature, GTR, while preserving or improving neurological function, is primarily the optimal treatment of choice. Significant removal of bony structures and ligaments is often required to access an operable surgical field. However, gaining access to the spinal cord through the spine can create postoperative spinal instability if not minimised [72,73]. As a result, surgeons often must decide on either open or minimally invasive approaches to their operation.

### 4.1. Open vs. Minimally Invasive Surgery

Access to the IDEM tumours is most commonly performed through posterior total laminectomy [74,75]. This is an open surgical approach that involves removing the whole lamina with the aim of creating a sizeable surgical field, making resection of the tumour easier both visually and mechanically. Articles included in this review that operated using this approach found positive results, of which six were able to fully resect 100% of the tumour with minimal recurrence and death [18,24,26,36,37]. However, we found this approach resulted in several intraoperative and postoperative complications, most notably CSF leakage and new neurological deficits [12,17,18,19,24,25,26,28,30,31,32,33,35,36,37,38]. In addition, as this method involves the creation of a large osseo-ligamentous defect, it increases the likelihood of spinal instability and kyphosis [74,76].

Increasingly more surgeons are opting for minimally invasive approaches. The minimally invasive unilateral hemilaminectomy approach has shown to be a highly effective technique for IDEM tumour resection among the papers included [16,19,21,22,23,26,27,28,29,30,33,34]. The consensus is that minimally invasive approaches drive refinement of the operative technique, allowing for greater anatomical appreciation and thus reducing intraoperative blood loss and complications thanks to a smaller durotomy and the potential preservation of the contralateral osseo-ligamentous structures [26,28,30,33]. Therefore, patients can recover quickly and be discharged earlier [26,30]. There is also a suggestion that minimally invasive approaches allow for more complete resections due to the use of higher magnifications, which provide a more precise field of vision [30]. Although no noticeable difference in the rate of GTR was noted between the studies in the minimally invasive group compared to the open surgery group, we did note substantially fewer reported complications. Interestingly, three authors stated that no complications were observed in their study, which was reported only once in the open surgery papers [22,26,29].

Whilst a minimally invasive approach can produce similar results to open surgery with fewer complications, it requires specialised training and experience to ensure adequate visualisation and maximise the extent of resection [75,77]. Moreover, long or more extensive tumours may necessitate additional laminectomy. Despite these considerations, minimally invasive methods show excellent potential over open surgical approaches in reducing rates of short- and long-term complications of IDEM surgery.

### 4.2. Surgical Approach

The surgical route largely depended on tumour location among the included articles. As most IDEM tumours are located posteriorly or laterally, surgeons approach them posteriorly in most cases. However, surgeons noted particular difficulty in managing anteriorly located tumours. Anterior tumours located in the thoracic or lumbar regions were almost entirely approached posteriorly due to substantial problems associated with an anterior approach [16,20,27,32,37]. However, surgeons would often adapt their approach to the anterior, for example, by extending incisions laterally or dissecting the dentate ligament to avoid spinal cord damage [16,27,30,31,32]. Moreover, a recent scoping review has demonstrated the effectiveness of the Da Vinci Robot for approaching thoracic or lumbar regions anteriorly [78]. Cervical tumours, on the other hand, can be resected either anteriorly or posteriorly depending on which side the tumour is located, often taking a transoral, transcervical, or transthoracic approach. Nevertheless, Acosta Jr et al. focused entirely on a transpedicular anterior approach for ventrally located tumours in the cervical or cervicothoracic region and demonstrated great success with no reported complications; thus, it could be a more optimal approach for anteriorly located rostral tumours [37].

### 4.3. Prognosis

Prognosis can vary considerably in IDEM tumours, and several factors can influence it. As with most tumours, prognosis heavily relies on the extent of resection. When GTR is achieved, patients generally have an excellent prognosis, with neurological improvement, improved quality of life, and low recurrence [12,13]. As a result, the prognosis in IDEM is relatively good due to the high rates of GTR and benign nature with minimal recurrence and mortality. Tumours ventral to the spinal cord tend to be more challenging to operate on, particularly in thoracic and lumbar lesions. They can result in higher rates of subtotal resection and, thus, a worse prognosis [8,16,37,79].

Historically, it has been noted that worsened prognosis tended to occur due to preoperative symptoms and surgical difficulty—longer preoperative duration and severity of symptoms significantly reduced prognosis [80,81]. For example, patients presenting preoperatively with paraplegia or quadriplegia often have a relatively poor neurological prognosis [79]. Despite this, among all our included articles, we found that several authors reported greatly improved function following surgery, with particularly significant improvements in patients who had worse performance grades preoperatively [12,16,17,19,23]. In addition, only one death was reported among the assessed studies related to the IDEM tumour [19].

When comparing prognosis in open versus minimally invasive papers, there was a lack of documents providing details on performance outcomes and variability in the performance scale used, which made it challenging to compare performance differences between the surgical techniques. Despite this, one study comparing both open and minimally invasive approaches did state that they found no significant differences in postoperative ASIA scores between the two methods.

Aside from performance outcomes, we did note several more cases of recurrence in the open surgery group among the studies [19,20,25,28,32]. In comparison, only one study reported recurrence in the minimally invasive group [28]. There is no clear explanation as to why there would be higher recurrence rates among the open surgery group. From a surgical point-of-view, one would expect the opposite outcome due to open surgery providing a better field of view and, thus, better visualisation of whether GTR was achieved. Instead, this is likely due to the open surgery group having a much larger cohort of patients (1141 vs. 230 patients) and, therefore, a higher probability of recurrence among the group. Nevertheless, it would be interesting to see more institutions comparing open versus minimal IDEM surgery recurrence rates to confirm this finding.

### 4.4. Limitations

When conducting this systematic review, we found many papers with data on IDEM tumours but did not separate these results from those of other spinal tumours. As a result, this limited the number of cases we could gather, introducing a degree of selection bias. In this systematic review, we had significantly more patients undergoing open surgical procedures than minimally invasive procedures. Consequently, certain aspects such as complication rates, recurrences, and deaths will be higher in the group with more cases. The lack of standardisation between studies made comparing data difficult. This was particularly true for functional grading systems, where papers often used different grading systems that did not consistently assess the same criteria. Finally, as the mean duration of follow-up varied significantly between studies, long-term outcomes could not be analysed in this review.

## 5. Conclusions

IDEM tumours are rare tumours that remain challenging to manage. Much of our understanding relies heavily on case series and building on others’ experiences. Resection of the tumour should be provided at earlier stages of symptoms to provide the best possible prognosis. Whilst open surgery does produce the required GTR and neurological improvement, it is, therefore, a perfectly viable option. There is increasing evidence that minimally invasive surgery can provide the same rates of resection with potentially fewer complications. This, in turn, results in fewer postoperative costs and quicker discharges, making minimally invasive approaches cheaper and more beneficial for hospitals. Nevertheless, these findings should be interpreted cautiously, given the lower number of minimally invasive cases in this systematic review.

## Figures and Tables

**Table 1:** IDEM tumour pathologies across patients.

Tumour Pathology	Number of Patients
Schwannoma	1300 (49.0%)
Meningioma	922 (34.7%)
Ependymoma	94 (3.5%)
Neurofibroma	82 (3.1%)
Metastatic tumour	54 (2.0%)
Cyst (epidermoid/dermoid/arachnoid)	46 (1.7%)
Paraganglioma	19 (0.7%)
Lipoma	10 (0.4%)
Neurinoma	5 (0.2%)
Inflammatory tumour	3 (0.1%)
Ganglioma	3 (0.1%)
Hemangioblastoma	2 (0.1%)
Solitary fibrous tumour	1 (0.04%)
Chondroma	1 (0.04%)
Hemangioma	1 (0.04%)
Cellular myopericytic neoplasm	1 (0.04%)
Others/unspecified	112 (4.22%)
Total (patients)	2656

**Table 2:** IDEM tumour locations for patients undergoing open and minimally invasive surgery.

Tumour Location	Open (%)	Minimally Invasive (%)
Conus medullaris	32 (0.9%)	0 (0%)
Cervical	771 (20.8%)	121 (17.5%)
Thoracic	1523 (41.2%)	321 (46.5%)
Lumbar	1183 (32.0%)	184 (26.6%)
Sacral	7 (0.2%)	3 (0.4%)
Junctional	185 (5.0%)	62 (9.0%)
Total (patients)	3701	691

**Table 3:** IDEM tumour positions for patients undergoing open and minimally invasive surgery.

Tumour Position	Open (%)	Minimally Invasive (%)
Ventral	155 (22.3%)	13 (3.5%)
Ventrolateral	46 (6.6%)	85 (23.0%)
Dorsal	186 (26.8%)	42 (11.4%)
Dorsolateral	169 (24.3%)	164 (44.4%)
Lateral	139 (20.0%)	4 (1.1%)
Other	0 (0%)	61 (16.5%)
Total (patients)	695	369

**Table 4:** Surgical techniques employed in minimally invasive surgery cohorts.

Surgical Technique (Minimally Invasive)	Number of Patients
Non-tubular endoscopic	86 (11.5%)
Non-tubular microscopic	305 (40.7%)
Tubular endoscopic	21 (2.8%)
Tubular microscopic	262 (34.9%)
Unspecified	76 (10.1%)
Total	750

**Table 5:** Demographics and tumour pathologies of tubular endoscopic and non-tubular microscopic cohorts.

	Tubular Endoscopic Surgery	Non-Tubular Microscopic Surgery
Total patients	21	305
Mean age	23.3	21
M (%)	9	155
F (%)	12	150
Tumour pathology		
Schwannoma (%)	9 (47.4%)	154 (54.8%)
Meningioma (%)	6 (31.6%)	90 (32.0%)
Ependymoma (%)	0 (0%)	5 (1.8%)
Neurofibroma (%)	0 (0%)	3 (1.1%)
Cyst (%)	3 (15.8%)	0 (0%)
Others (%)	1 (5.3%)	29 (10.3%)

## Data Availability

The raw data supporting the conclusions of this article will be made available by the authors upon request.

## Abbreviations

The following abbreviations are used in this manuscript:

ASIA	American Spinal Injury Association impairment scale
CSF	Cerebrospinal fluid
GTR	Gross Total Resection
IDEM	Intradural-extramedullary
IONM	Intra-operative Neurophysiological Monitoring
PRISMA	Preferred Reporting Items for Systematic reviews and Meta-analyses
WHO	World Health Organisation
